# Teacher’s Perspectives About Tobacco Consumption and Its Prevention Among Students From Western Maharashtra, India: A Qualitative Study

**DOI:** 10.7759/cureus.45924

**Published:** 2023-09-25

**Authors:** Umesh Kawalkar, Shounak Joshi, Ashwini Patekar, Priti Kogade, Sampda Rajurkar, Shital Telrandhe

**Affiliations:** 1 Community Medicine, Government Medical College (GMC) Akola, Akola, IND; 2 Research and Development, Jawaharlal Nehru Medical College, Datta Meghe Institute of Higher Education and Research, Wardha, IND

**Keywords:** school, cigarettes and other tobacco products act (cotpa), qualitative study, adolescent, tobacco

## Abstract

Background

Teachers are role models for students and influential members of society. They are capable of influencing social norms related to tobacco control among students. This background study was planned to understand teachers’ opinions and views about factors influencing tobacco use and prevention strategies being used.

Methodology

We conducted qualitative research on teacher’s opinions about tobacco use among students. We chose focus group discussion as our data collection method, as we wanted to observe their personal views on social norms. We involved 70 high school teachers in our study from the Akola district. The data collected from the teachers were organized into various themes interrelated to the objectives.

Results

The majority of teachers mentioned that the reason for tobacco consumption among children was peer pressure, from observations of older individuals in society, and from TV serials. Some teachers suggested that proper counseling and telling them about the harmful effects of tobacco are useful for prevention. Tobacco’s harmful effects and its prevention strategies were not included in the standard curricula of students, which is one of the key barriers.

Conclusions

There is a need to implement school-based tobacco prevention education programs to reduce the early onset of smoking among students. School authorities must implement the Cigarettes and Other Tobacco Products (Prohibition of Advertisement and Regulation of Trade and Commerce, Production, Supply, and Distribution) Act, 2003 effectively with the help of the police to prohibit tobacco use among teachers and students.

## Introduction

Tobacco use is one of the foremost causes of chronic disorders such as oral cancer, cardiovascular diseases, pulmonary diseases, and stroke [1]. In the 20th century, it was noticed that there were 100 million premature deaths globally, and if this trend follows, there would be around 1 billion premature deaths in this century [2]. India is the biggest buyer and maker of tobacco. Tobacco is consumed in a variety of forms such as pan, pan masala, gutkha, and mishri, including smoking and chewing forms [3]. According to the Global Adult Tobacco Survey done in 2016-2017, nearly 28.6% of adults currently consume tobacco either in smoke or smokeless form, with men (42.4%) consuming more than women (14.2%) in India [4]. Around 90% of adult smokers start the habit in their youth. The Global Youth Tobacco Survey stated that 14.6% of Indian adolescents consumed tobacco products. India is right now home to the world’s biggest youth populace [5].

Prevention of early tobacco addiction in youth is an important step for reducing the burden of non-communicable diseases in the world [6]. Children and youth are considered more receptive to tobacco education than adults. Several programs have been implemented to curb tobacco usage among teenagers and school-going children. Sustainable and effective tobacco addiction prevention programs for youth are an ethical imperative. Schools are perfect settings for all children and adolescents for such types of programs [7]. The Government of India has developed dedicated guidelines and national legislation for the prevention of tobacco use. Cigarettes and Other Tobacco Products Act (COTPA Act 2003), and Tobacco-Free Educational Institutions (ToFEI) guidelines should be implemented strictly in schools; however, it is a very complex and challenging process [8].

Teachers are role models for students and influential members of society. Their influence extends beyond the classroom and can significantly impact a child’s understanding of the risks associated with tobacco use and the development of healthy behaviors [9]. Teachers play an important role in children’s tobacco prevention strategies by providing education and awareness about the risks of tobacco use, setting positive examples, teaching communication and refusal skills, creating a supportive environment, integrating tobacco prevention into the curriculum, utilizing resources and guest speakers, monitoring for signs of tobacco use, encouraging critical thinking, supporting at-risk students, and engaging in continuous professional development. Their influence extends beyond the classroom, helping to shape a tobacco-free generation and foster healthy behaviors among students [10,11]. This background study was planned to understand teacher’s opinions and views about factors influencing tobacco use and the prevention strategies being used.

## Materials and methods

A qualitative study was conducted at different government, government-aided, and private schools in Akola district, Maharashtra, India. All teachers involved in teaching 8th to 12th-grade students (13 to 17 years of age) were included in the study. A total of 10 schools participated in the study. There were a total of 98 teachers in all schools. In total, 70 teachers participated in the study and 12 refused to participate in the study. Teachers with experience of more than one year who were willing to participate in the study were included. Nine teachers were excluded from the study. Others were on leave on the day of data collection. A purposive sampling strategy was used to recruit study participants. We chose focus group discussion (FGD) as our data collection method. A total of seven FGDs were conducted in schools based on the data saturation method. The FGD guide was designed with nine open-ended questions seeking information about the teacher’s perception of tobacco consumption among students and factors influencing the prevention strategies for tobacco consumption and to identify resources and support for effective implementation of tobacco control interventions within schools (Appendices). The FGDs, which lasted between 50 and 75 minutes, were conducted in the local language. An average of 9-11 respondents participated in each FGD, and, overall, there were 70 respondents. The sessions were moderated by a facilitator who was trained to conduct FGDs among teachers as well as a note taker to document the verbal responses and non-verbal cues during the discussions. Facilitators and note-takers were postgraduate students from the Department of Community Medicine. FGDs were supplemented with the use of a tape recorder. Informed consent was obtained from each participant. We understood and ensured the confidentiality of the FGD, and encouraged participants for their opinion. Government Medical College Akola Institutional Ethics Committee gave approval for conducting the study (approval number: GMCA/IEC Anat/9206/2022).

All answers recorded were transformed into English transcripts, which were then coded using descriptive words or phrases manually by AP and PK. Coded transcripts were organized for emerging themes manually by AP and PK and reviewed by SR and ST for consistency checks and rigor. Data were collected under the themes of the perception of teachers regarding the reasons for tobacco use among students, knowledge of the health hazards of tobacco use among teachers, and inclusion of prevention strategies in the curriculum. The outline of themes was reviewed and edited as needed. Data are presented textually with quotes to illustrate study findings.

## Results

A total of 70 teachers belonging to 10 different schools were involved in the study. Among those, 19 were from government schools, 23 were from government-aided schools, and 28 were from private schools in Akola. Out of the 70 participants, 15 (21.4%) were female and 55 (78.5%) were male. The average years of teaching experience (SD) among study participants was 11.4 (±5.6) years (Table 1).

**Table 1:** Sociodemographic variables of study participants.

Variable	Number (%)
Age (mean ± SD)	46.4 (±6.9)
Male:Female	15:55
Work experience (years)	
<10 years	27 (23.3%)
>10 years	43 (76.7%)

The data collected were organized into themes related to the objectives of the study (Table 2).

**Table 2:** Categorization of perception of study participants.

Theme	Subtheme
Perception of teachers regarding reasons for tobacco use among students	Parent’s habit of tobacco consumption; poor economic conditions of family; friends and friend circles; use of tobacco in movies and TV serials; easy availability of tobacco products
Knowledge about ill health related to tobacco use	Only specific health consequences such as oral cancer; unaware about other health effects other than oral cancer; lack of knowledge about the available resources and services for tobacco deaddiction
Tobacco addiction among teachers	No one should consume tobacco and tobacco-related products in front of students
Preventive strategies for tobacco use	Not included in the school curriculum; taught once in a semester in life skill classes; basic message and teaching have only a temporary impact; need to teach about how to handle peer pressure; various methods of teaching should be used; implementation of the COTPA Act at the school level with help of other stakeholders; education sessions for parents

Perception of teachers regarding the reason for tobacco use among students

Teachers expressed concern that students take up the habit of tobacco from their parents or the elderly nearby. The reason was the unawareness and lack of education about the hazards of tobacco use, especially for their children. The other reason can be poor economic conditions, leading both parents to work giving them less time to monitor the habits and company of their children. As one of the participants rightly expressed “Students easily follow the elderly. He saw many adult people surrounding him consume tobacco and smoke. He is also curious about it.” They also mentioned that the reason for tobacco consumption among children was peer pressure. Friend and friend circles make a great impact on a student to take up a habit. Another influencing factor was the use of tobacco in movies, TV serials, and advertisements. The reason can be the curiosity among the children to try it for fun or to imitate the one seen in TV advertisements. Increased sales in the market reflect the easy availability of tobacco products. This might lead to increased use among the youth and children and make them addicted to it.

Knowledge about ill health related to tobacco use

Teachers were unaware of the deleterious health effects of tobacco use, with the majority of them being unable to tell specific health consequences such as oral cancer. Only a few teachers knew about other effects of tobacco use such as oral health. Teachers were found to have more knowledge about the ill-health effects of smoking than that of the chewable forms of tobacco. Teachers also knew about the deleterious effects of passive smoking on individuals along with active smoking. Some teachers reflected on the lack of certainty about health problems related to tobacco usage. Consideration of only carcinogenic effects and unawareness about other health impacts including premalignant conditions was the leading cause for them to think about the certainty of ill effects on health. One teacher said “Some people who consume tobacco remain healthy even after a long time. It is not that everyone gets cancer if they consume tobacco.” It has been also observed that teachers lacked knowledge about the available resources and services for tobacco deaddiction. There is a need to create awareness about the available de-addiction resources to help people get rid of tobacco addiction.

Tobacco addiction among teachers

Almost all teachers strongly agreed that no one should consume tobacco and tobacco-related products in front of students. Few teachers were reluctant to respond as they were unwilling to admit the truth about the consumption of tobacco in front of students or they were possibly hiding their use of tobacco products. One teacher said “Teachers are next to parents of child. They follow us so we must not set a bad example among them. So, we are compelled ethically not to eat tobacco at any time in front of them.”

Preventive strategies for tobacco use among students

Students are taught strictly according to the curriculum set by the state or central governments. Tobacco prevention is generally not included in the school curriculum expect for a few chapters included in the moral sciences subject. According to one of the teachers, “the curriculum is itself so vast that no light can be put over the issues of bad habits or addiction.” The part of moral education has the least importance in academics with most teachers skipping the chapter. A teacher mentioned that tobacco use prevention is taught once in semester in life skill classes. Teaching includes the basic message that “don’t use tobacco; it is bad for your health.” This teaching has only a temporary impact. Few teachers stated that tobacco prevention issues should be covered broadly. Along with the health hazards, there is a need to teach students about how to handle peer pressure which is a major cause for teens to fall for such habits. Moreover, they should be taught to “say no” to such habits. They need to be guided regarding curiosity toward tobacco and tobacco-related products. Various methods such as standard classroom activities like lectures, lectures along with demonstrations, role-play activities, and case studies should be used to teach tobacco prevention activities. Teachers also expressed the need for implementation of the COTPA Act at the school level. Teachers also reported that non-teaching staff, police, and social activists can be involved. Education sessions for parents can also be useful for the purpose.

## Discussion

In this qualitative study, various factors related to the use of tobacco among the youth in view of the teachers were studied. According to the Global Adult Tobacco Survey, the prevalence of tobacco use increased among the adolescent age group. Hence, to understand the issues and views among teachers teaching students studying in the 8th to 12th grades were included in the study [12]. Among the various factors responsible, the well-known factor of peer pressure was discussed by the teachers. Teachers considered peer pressure and the friend circle as leading causes for the students to take up the habit. The association between smoking habits and peers has been well studied. A review of qualitative research assessed peer pressure along with interpersonal influences on smoking. Smoking behaviors among adolescents are influenced at socioecological levels [13]. Adolescent smoking has been related to characteristics such as family stressors, deprived family relations, low self-esteem, low school satisfaction, and poor grades in school [14,15].

In this study, teachers expressed concern that students take up the habit of tobacco from their parents or the elderly nearby. The reason was the lack of education and working parents. A study conducted by Nichter et al. and Narayan et al. also mentioned similar findings that smoking by friends and family members are important factor among children for tobacco use [16,17]. Lack of knowledge among the parents highlights the role of tobacco education programs among parents along with the students. The parents must be knowledgeable about the harms of tobacco and they themselves should not be addicted to tobacco. Restriction by parents provides children with limited access to such products. Considering parents’ role in tobacco cessation, workshops for parents should also be organized in schools and other social activities should be carried out [12]. The role of role models, i.e., TV serials and advertisements promoting tobacco, is also an important factor. In our study, teachers were concerned about the use of tobacco in movies, TV serials, and advertisements, which are also important influencing factors. Media advertisements influence children’s perception and initiation of tobacco use, as reported by studies conducted by Sreeshyla et al. and Gururaj et al. [18,19]. The study conducted by Kulkarni et al. mentioned that tobacco content was common in films of national languages. It was not suitable for viewing by children. National tobacco control laws were not complied with fully in films. Strict enforcement of tobacco-free film rules is needed so that exposure to tobacco use on the screen will not be shown to the child which will protect them [20].

Considering all these factors, the effort for tobacco prevention needs to be put in various ways. As the teachers suggested changes need to be made in the school curriculum. Stress should be given on healthy habits and the topic of tobacco should be compulsorily taught each year. This includes teaching them how to resist peer pressure, to “say no” to such habits, health education workshops, and activity participation of students in social activities condemning tobacco usage. Implementation of the COTPA Act in every school should be done with the help of all teaching as well as non-teaching staff. The COTPA Act banned all forms of advertising of tobacco products [21]. Proper implementation of this act has successfully reduced the prevalence of tobacco use (ban on sales to underage persons and ban on selling outlets within 100 yards of an educational institution). Multi-sector approach including other departments such as police and health will be needed for the effective implementation of tobacco control activities [12].

## Conclusions

Tobacco use prevention in schools needs a multi-pronged approach including proper policymaking, teacher training and prohibition of tobacco use by teachers, the inclusion of tobacco prevention and cessation-related knowledge in the school curriculum, motivation drive, parent’s education, and awareness programs by including other non-teaching staff, police, and social workers. It is also recommended that school programs should be more than curriculum teaching to teach them about life skills to refuse tobacco. There should be essential infrastructure support for these tobacco prevention activities. The school authority must implement the COTPA Act effectively with the help of the police department including prohibiting tobacco use among teachers and students. Teachers should play a proactive role in tobacco prevention by integrating education, promoting critical thinking, and being supportive. They should lead by example, encourage discussions, and organize awareness campaigns, fostering a tobacco-free environment. Through education, counseling, and advocacy for policies against tobacco use, teachers actively contribute to students' well-being. Their commitment to monitoring and reporting signs of tobacco use ensures a healthier school community.

## Appendices

Focus group discussion questionnaire

Section A (Reason for Tobacco Consumption Among Students)

1. Are you aware of the health risks and consequences associated with tobacco use? (Please elaborate).

2. Are you aware of any students in your school who use tobacco products? Please specify the types of tobacco products they use.

3. In your opinion, what are the primary causes or reasons behind students’ tobacco consumption in school?

4. Could you describe the personal approach to addressing tobacco-related issues with your students? Have you encountered any challenges in this regard?

5. What is your opinion regarding tobacco addiction among teachers in school or the educational community?

Section B (Preventive Strategies Against Tobacco Consumption)

6. In your opinion, what role should schools play in preventing tobacco consumption among students?

7. Are there any existing tobacco prevention programs or activities in your school that you think have been particularly effective? Please describe them.

8. What suggestions do you have for improving tobacco prevention efforts in schools?

9. Do you have any additional comments or insights on the topic of tobacco consumption and prevention among students?
